# Extrinsic and Intrinsic Competition between *Chouioa cunea* Yang and *Tetrastichus septentrionalis* (Hymenoptera: Eulophidae), Two Pupal Parasitoids of the Fall Webworm, *Hyphantria cunea* (Lepidoptera: Erebidae)

**DOI:** 10.3390/insects15080617

**Published:** 2024-08-15

**Authors:** Zhixin Li, Liyuan Yang, Xi Ma, Xudan Liu, Yiran Cheng, Shouhui Sun

**Affiliations:** College of Forestry, Shenyang Agricultural University, Shenyang 110866, China; zhuti0225@163.com (Z.L.); yangliyuan_good@126.com (L.Y.); xy2297741062@163.com (X.M.); 13009239409@163.com (X.L.); czlhcl@gmail.com (Y.C.)

**Keywords:** multiparasitism, time interval, endoparasites, offspring quality

## Abstract

**Simple Summary:**

Competition arises for the monopolization of host resources when multiple parasitoid species attack the same host. This study focused on the competition between *Chouioa cunea* Yang and *Tetrastichus septentrionalis* Yang (Hymenoptera: Eulophidae), two pupal parasitoid species of the fall webworm, *Hyphantria cunea* (Drury) (Lepidoptera: Erebidae). Experiments were conducted to investigate extrinsic and intrinsic competition. The results showed that both parasitoid species were capable of parasitizing hosts that were already parasitized by the competitor, with the first released species demonstrating superiority as an intrinsic competitor in multiparasitized hosts across various parasitoid release orders and time intervals between oviposition. These findings contribute to a better understanding of the interactions between the two parasitoids within the host pupae and offer insights into the biological control of *H. cunea* and other leaf-eating pests.

**Abstract:**

The endoparasitoids *Chouioa cunea* Yang and *Tetrastichus septentrionalis* Yang (Hymenoptera: Eulophidae) are both gregarious pupal parasitoids of the fall webworm, *Hyphantria cunea* (Drury) (Lepidoptera: Erebidae). In order to analyze the competitive interactions between both parasitoids exploiting *H. cunea*, we assessed both extrinsic and intrinsic competition. The search time, oviposition duration, and oviposition frequency were used as evaluation criteria for extrinsic competition. The number of survival days, female ratio, and number of parasitoids emerging from the host were used as evaluation criteria for intrinsic competition. The results indicated that both parasitoid species were able to parasitize hosts that were already parasitized by competitors. The first released species consistently emerged as the superior competitor in multiparasitized hosts. Both parasitoid release orders and time intervals between oviposition affect the competition of parasitoids and the parasitic efficiency. The results emphasize the parasitic abilities of both parasitoid species and provide a basis for future research on competition mechanisms and biological control of *H. cunea*.

## 1. Introduction

*Hyphantria cunea* (Drury) (Lepidoptera: Erebidae), the fall webworm, is native to North America. In its native habitat, *H. cunea* does not pose a significant threat. However, its introduction to Asia and Europe has resulted in notable destructive capabilities [1,2]. This species has a broad range of hosts and rapid reproduction rates, and causes substantial damage to agricultural and forest ecosystems [3]. Since the species invaded China, the occurrence of *H. cunea* has caused serious disturbance to the ecological construction of forestry, leading to more than 40 years of endeavors to explore effective control measures [4,5]. Despite advancements in control methods, challenges still persist [6]. Amid these challenges and opportunities, the advantages of parasitic wasps are exploited.

Hymenoptera, one of the four mega-diverse insect orders, plays a fundamental role in virtually all terrestrial ecosystems, as parasitoids, predators, and pollinators, and is of considerable economic value [7,8]. Among them, parasitoids are considered to be the most important biological control agents [9]. *Chouioa cunea* Yang and *Tetrastichus septentrionalis* Yang (both Hymenoptera: Eulophidae) are koinobiont pupal endoparasitoids of *H. cunea*, initially collected in wild ecosystems [10]. *C. cunea* has been collected in various provinces in China, including Shanxi, Liaoning, Hebei, and Shandong, as well as in countries such as Japan and Italy [11]. This species effectively parasitizes a wide array of lepidopteran insects, being native to East Asia and introduced to different parts of the world [12]. *T. septentrionalis* is one of the dominant pupal parasitic natural enemies of *H. cunea* in Dandong, Liaoning Province, China. It has the ability to parasitize the pupae of various leaf-eating pests such as *Stilpnotia candida* Staudinger and *Stilpnotia salicis* (Linnaeus) (both Lepidoptera, and Lymantriidae), showing potential for biological control of *H. cunea* [13].

Reproductive parasitism by hymenopteran insects is diverse and complex [14]. Existing statistics indicate that releasing multiple parasitoids versus a single parasitoid can significantly increase the mortality of the biocontrol agents and reduce the pests’ abundance [15]. However, the introduction of multiple parasitoids may disrupt biological control mechanisms and increase competition between parasitoids, reducing the effectiveness of parasitoids in controlling target pests [16,17]. The competition of the parasitoids could be mainly divided into extrinsic and intrinsic competition. Extrinsic competition covers the time and ability of the searching host, while intrinsic competition explores attributes associated with immature parasitoids developing on or inside the host [18]. Therefore, it is important for biological control programs to consider the interactions between parasitoids [19,20]. More commonly, each species of parasitoids has no inherent advantage and there is competition between them. The superior completes its development, while the inferior dies in the form of eggs or larvae [16].

Several previous studies have assessed the interactions among parasitoids targeting leaf-eating pests in agriculture and forestry [21,22], but there is a lack of research on the interactions between parasitoids of *H. cunea*. In our preliminary survey, we found these two dominant parasitoids in Dandong City, Liaoning Province, China. During our field investigation, we observed pupae collected from sample spots infested by *H. cunea*. None of the pupae collected from the sample spots yield two species of parasitic wasps simultaneously. Ascertaining the factors responsible for this outcome could be investigated by competitive experimental approaches.

Despite the implementation of biological control measures in China, the effectiveness and extent of prevention through monoparasitism still fail to reach the level conducive to sustainable management of pests. Therefore, we focused on the interaction between two species of pupal parasitoid of *H. cunea*, in order to explore the potential for combined biological control of *H. cunea* by using two species. Parasitoid release orders and time intervals between oviposition significantly impact the parasitism outcome. Thus, we hypothesized that: (i) both extrinsic and intrinsic competition can affect the parasitic efficiency of parasitoid; (ii) the first released parasitoid species is superior, taking advantage of intrinsic competition. Moreover, when endoparasitoids parasitize the same host, usually only one species of the offspring emerges from the host. Therefore, we assessed: (iii) when two parasitoid species parasitize the same host, only one parasitoid could eventually emerge.

## 2. Materials and Methods

### 2.1. Insect Rearing

*Chouioa cunea* and *Tetrastichus septentrionalis* were collected from the pupae of *H. cunea* in Dandong City, Liaoning Province (N 40°5′22″, E 124°20′26″). As male parasitoids can mate with multiple female parasitoids, parasitoids were placed in a 10 mL centrifuge tube at a male-to-female ratio of 1:10. Absorbent cotton containing 10% honey water was added to provide nutrition. After adequate mating, both parasitoid species were reared and cultured as follows: one pupa of *H. cunea* and three female wasps were placed in a 10 mL centrifuge tube in a laboratory setting incubators (RXZ-260A, Ningbo Dongnan Instrument Co., Ltd., Ningbo, China) at 24 ± 1 °C, 65% ± 5% RH and 16 L:8 D. Since parasitism capability of these two species may not be maintained under controlled conditions of laboratory for long time periods, the experiments included parasitoids within three generations. We used cotton with 10% honey water to provide nutrition for parasitoids. Both of the parasitoid species were identified by expert taxonomists.

*Hyphantria cunea* eggs were obtained from the Forest Ecological Environment and Protection Research Centre of the Chinese Academy of Forestry Sciences (Beijing, China) and reared by using artificial diets at 24 ± 1 °C, 65% ± 5% RH, and 16 L:8 D until pupation. In order to eliminate the influence of the host quality on parasitism, the pupae of *H. cunea* used in the experiment were all within three days of pupation.

### 2.2. Extrinsic Competition: The Order of and Time Intervals between Parasitism by Two Pupal Parasitoids

Based on the previous research on the developmental duration of *C. cunea* and *T. septentrionalis* [23,24], we set up the experimental groups of solitary parasitism and co-parasitism (control: *C. cunea* alone, *T. septentrionalis* alone, and both *C. cunea* and *T. septentrionalis* parasitism simultaneously) and three different time intervals between oviposition by two species and orders: *C. cunea* released first and *T. septentrionalis* released first. Two parasitism sequences (species order: Cc-Ts and Ts-Cc) were used in the experiments, with variation in the time interval between attacks by the two parasitoid species. In the Cc-Ts sequence, a single *C. cunea* female parasitized one host pupa, which was later exposed to *T. septentrionalis* at 24 h, 48 h, or 72 h after the initial oviposition. In the Ts-Cc sequence, the same procedures with the same set-up of time intervals were applied. The female parasitoids after mating and nutritional supplementation (48–72 h old) were used in the experiments. A pupa of *H. cunea* was placed in the center of the 120 mm diameter Petri dish and a parasitoid was placed along the wall. Observations were made by using the Sh200 video detection system (Beijing Hengsanjiang Instrument Sales Co., Ltd., Beijing, China) through a stereo microscope (SMZ475T Nikon Instruments Co., Ltd., Shanghai, China) to record the search time, oviposition frequency, and oviposition duration of parasitoids (the species released later) for each treatment. The search time refers to the period starting from when the parasitoid begins searching until it locates the host and performs oviposition. Oviposition frequency is recorded as the instances of egg-laying by the parasitoid throughout the entire interaction with the host. Oviposition duration is the total time taken for all egg-laying processes by the parasitoid. All treatments were carried out at a temperature of 24 ± 1 °C, 65% ± 5% RH, and 16:8 L:D. Each treatment was repeated 10 times.

### 2.3. Intrinsic Competition: The Order of and Time Intervals between Parasitism by Two Pupal Parasitoids

Similar to the extrinsic competition, the intrinsic competition was set up for solitary parasitism and co-parasitism (control: *C. cunea* alone, *T. septentrionalis* alone, and both *C. cunea* and *T. septentrionalis* parasitism simultaneously) and three different time intervals between oviposition by two species and orders: *C. cunea* released first and *T. septentrionalis* release first. In this study, nutrients were provided to the parents used in the experiment. To prevent potential impacts of nutrients on the survival days of offspring, we did not supplement offspring with nutrients. We recorded the species, number, survival days and female ratio of parasitoid offspring after they emerged from the host by using a stereo microscope (SMZ475T Nikon Instruments Co., Ltd., Shanghai, China).

All parasitoid offspring were classified as *C. cunea* or *T. septentrionalis*. The identification of the two parasitoid wasps is mainly based on the size and antennae of the parasitoid itself: the size of the female wasp of *T. septentrionalis* is significantly larger than that of *C. cunea*, and the female antennal club section is obviously different. The number of parasitoid offspring is the sum of all female and male parasitoids. Survival days refer to the number of days from emergence to death for all parasitoids that emerged from the same host. To ensure consistency in nutrient intake among parasitoids, no additional nutrients were provided. Each treatment was repeated 25 times.

The equations calculating the female ratio of parasitoids, mortality rate, parasitism rate, and host emergence rate are as follows.

(1) $$R_{F}\left(\%\right)=\frac{N_{F}}{N_{a}}\times 100$$

where *R_F_* represents the female ratio of parasitoid offspring, *N_F_* represents number of female parasitoids, and *N_a_* represents number of all pupae.

(2) $$R_{M}\left(\%\right)=\frac{N_{a}-N_{P}-N_{H}}{N_{a}}\times 100$$

where *R_M_* represents mortality rate, *N_a_* represents number of all pupae, *N_P_* represents number of successfully parasitized pupae, and *N_H_* represents number of host-emerged pupae.

(3) $$R_{P}\left(\%\right)=\frac{N_{P}}{N_{a}}\times 100$$

where *R_P_* represents parasitism rate, *N_a_* represents number of all pupae, and *N_P_* represents number of successfully parasitized pupae.

(4) $$R_{H}\left(\%\right)=\frac{N_{H}}{N_{a}}\times 100$$

where *R_H_* represents host emergence rate, *N_a_* represents number of all pupae, and *N_H_* represents number of host-emerged pupae.

### 2.4. Statistical Analysis

Because experimental data did not meet the assumptions of a normal distribution and equal variances for parametric methods, even after transformation, we used a nonparametric one-way ANOVA (Kruskal–Wallis test) to compare the differences in extrinsic competition (search time, oviposition frequency, oviposition duration) and intrinsic competition (female ratio of offspring, number of offspring and number of survival days of offspring) at the different species parasitoid with the same parasitoid release modes (release orders and time intervals between oviposition) or with different parasitoid release modes at the same species parasitoid. When the Kruskal–Wallis test indicates that at least two groups differ significantly, Dunn’s test is subsequently employed for post-hoc multiple comparisons. For all tests, a threshold of *p* < 0.05 was used. Investigated were carried out with SPSS statistical software (v24, SPSS Inc., Armonk, NY, USA).

## 3. Results

### 3.1. Results of Extrinsic Competition: The Order of and Time Intervals between Parasitism by Two Pupal Parasitoids

**Figure 1:**
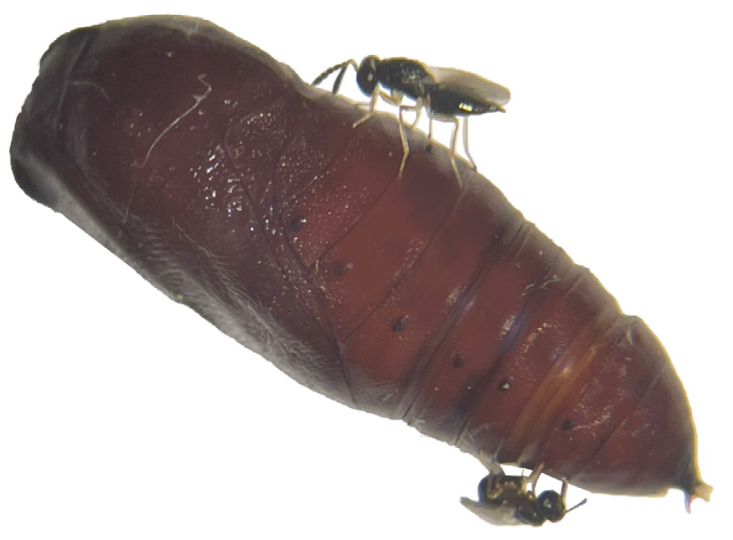
*Chouioa cunea* and *Tetrastichus septentrionalis* parasitize the same pupa of *Hyphantria cunea*. Annotation: upper side of the picture is *T. septentrionalis* and the lower side is *C. cunea*.

**Figure 2:**
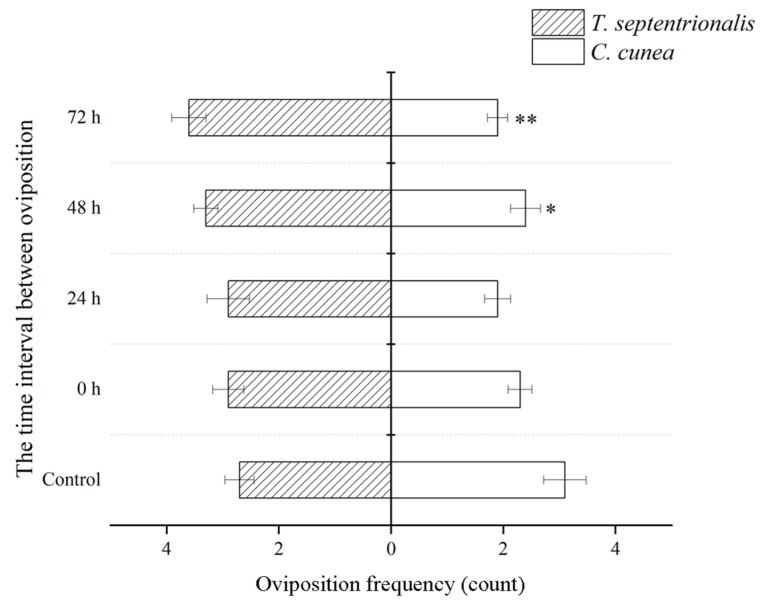
The differences in oviposition frequency between *Chouioa cunea* and *Tetrastichus septentrionalis* were observed in *Hyphantria cunea*. Annotation: Data are presented as mean ± SEM. Control, where only *C. cunea* or *T. septentrionalis* was released; 0 h, where *C. cunea* and *T. septentrionalis* were introduced simultaneously; Ts-Cc 24 h, Ts-Cc 48 h, and Ts-Cc 72 h, where *T. septentrionalis* was released 24 h, 48 h, and 72 h before *C. cunea*; Cc-Ts 24 h, Cc-Ts 48 h, and Cc-Ts 72 h, where *C. cunea* was released 24 h, 48 h, and 72 h before *T. septentrionalis*. * indicates a difference and ** indicates a strong difference (*p* < 0.05; *p* < 0.01). The number of repetitions is 10.

Both parasitoid species can lay eggs on the same host (Figure 1). Significant differences in oviposition frequency were observed among different species of parasitoids with the same release modes, showing significant variations at 48 h intervals and highly significant differences at 72 h intervals (Figure 2).

**Figure 3:**
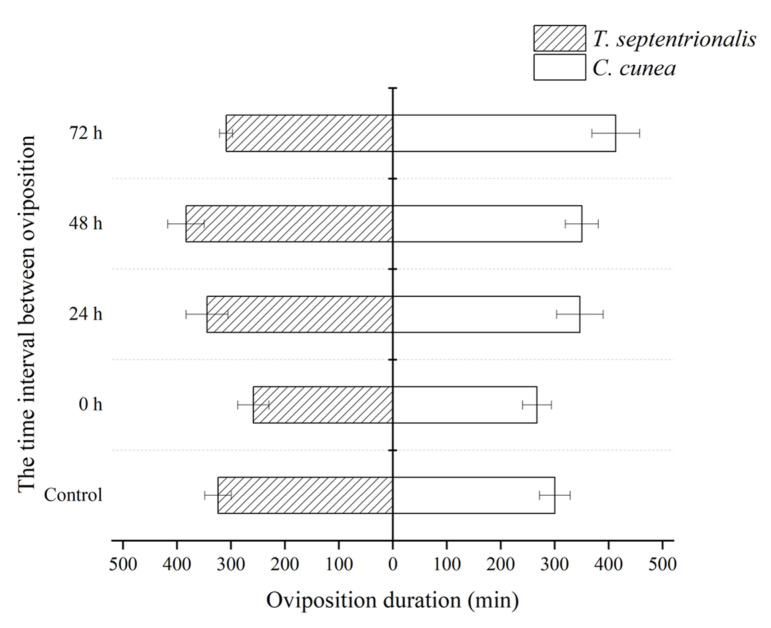
The differences in oviposition duration between *Chouioa cunea* and *Tetrastichus septentrionalis* were observed in *Hyphantria cunea*. Annotation: Data are presented as mean ± SEM. Control, where only *C. cunea* or *T. septentrionalis* was released; 0 h, where *C. cunea* and *T. septentrionalis* were introduced simultaneously; Ts-Cc 24 h, Ts-Cc 48 h, and Ts-Cc 72 h, where *T. septentrionalis* was released 24 h, 48 h, and 72 h before *C. cunea*; Cc-Ts 24 h, Cc-Ts 48 h, and Cc-Ts 72 h, where *C. cunea* was released 24 h, 48 h, and 72 h before *T. septentrionalis*. The number of repetitions is 10.

The oviposition duration was observed among different species of parasitoids with the same release modes, showing no significant difference (Figure 3).

**Figure 4:**
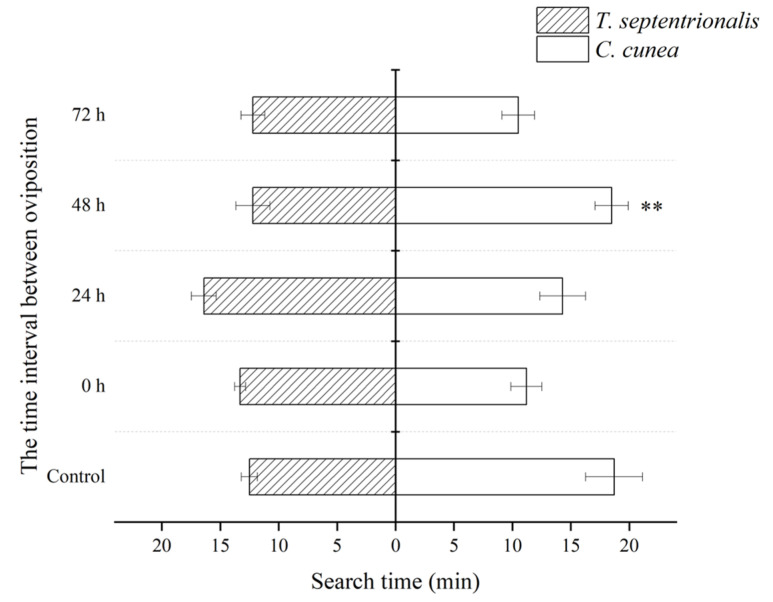
The differences in search time between *Chouioa cunea* and *Tetrastichus septentrionalis* was observed in *Hyphantria cunea*. Annotation: Data are presented as mean ± SEM. Control, where only *C. cunea* or *T. septentrionalis* was released; 0 h, where *C. cunea* and *T. septentrionalis* were introduced simultaneously; Ts-Cc 24 h, Ts-Cc 48 h, and Ts-Cc 72 h, where *T. septentrionalis* was released 24 h, 48 h, and 72 h before *C. cunea*; Cc-Ts 24 h, Cc-Ts 48 h, and Cc-Ts 72 h, where *C. cunea* was released 24 h, 48 h, and 72 h before *T. septentrionalis*. ** indicates a strong difference (*p* < 0.05). The number of repetitions is 10.

Highly significant variations in search time were noted among different species of parasitoids with the same release modes, showing highly significant variations at the 48 h interval (Figure 4).

### 3.2. Results of Intrinsic Competition: The Order of and Time Intervals between Parasitism by Two Pupal Parasitoids

**Figure 5:**
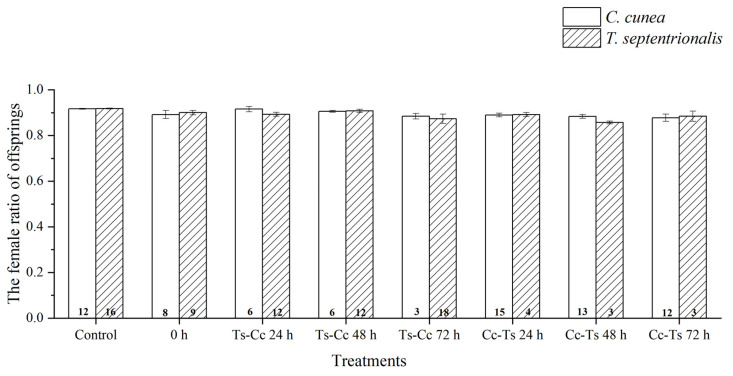
The differences in female ratio of offspring between *Chouioa cunea* and *Tetrastichus septentrionalis* were observed in *Hyphantria cunea*. Annotation: Data are presented as mean ± SEM. Control, where only *C. cunea* or *T. septentrionalis* was released; 0 h, where *C. cunea* and *T. septentrionalis* were introduced simultaneously; Ts-Cc 24 h, Ts-Cc 48 h, and Ts-Cc 72 h, where *T. septentrionalis* was released 24 h, 48 h, and 72 h before *C. cunea*; Cc-Ts 24 h, Cc-Ts 48 h, and Cc-Ts 72 h, where *C. cunea* was released 24 h, 48 h, and 72 h before *T. septentrionalis*. The values displayed at the base of each column indicate the number of replicates.

**Figure 6:**
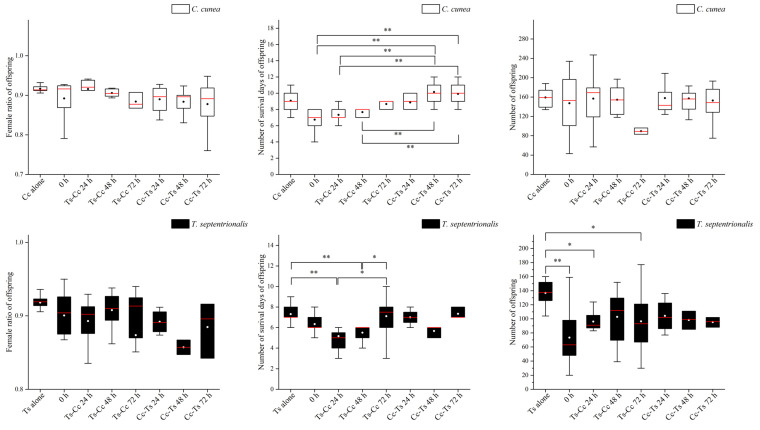
The differences in female ratio, number of survival days, and number of offspring of *Chouioa cunea* and *Tetrastichus septentrionalis* under the different conditions. Annotation: Data are presented as mean ± SEM. Cc alone, where only *C. cunea* was released; Ts alone, where only *T. septentrionalis* was released; 0 h, where *C. cunea* and *T. septentrionalis* were introduced simultaneously; Ts-Cc 24 h, Ts-Cc 48 h, and Ts-Cc 72 h, where *T. septentrionalis* was released 24 h, 48 h, and 72 h before *C. cunea*; Cc-Ts 24 h, Cc-Ts 48 h, and Cc-Ts 72 h, where *C. cunea* was released 24 h, 48 h, and 72 h before *T. septentrionalis*. * indicates a difference and ** indicates a strong difference (*p* < 0.05; *p* < 0.01).

The outcomes of parasitism by two parasitoid species varied under different conditions (Table 1). The female ratio of offspring did not exhibit significant differences between different species of parasitoids using the same release modes (including release orders and time intervals between oviposition), or between different release modes of parasitoids within the same species (Figure 5 and Figure 6).

**Figure 7:**
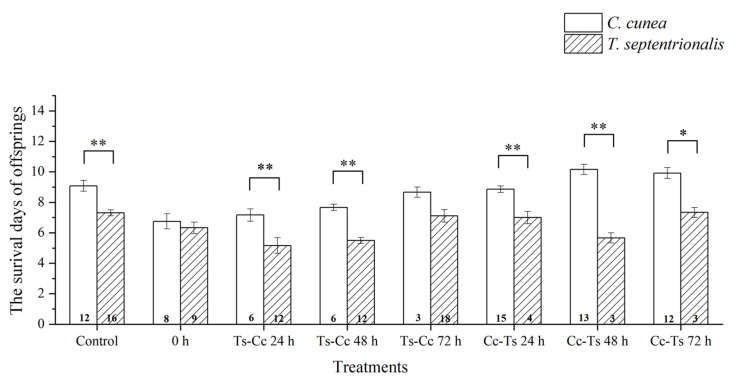
The differences in number of survival days of offspring between *Chouioa cunea* and *Tetrastichus septentrionalis* were observed in *Hyphantria cunea*. Annotation: Data are presented as mean ± SEM. Control, where only *C. cunea* or *T. septentrionalis* was released; 0 h, where *C. cunea* and *T. septentrionalis* were introduced simultaneously; Ts-Cc 24 h, Ts-Cc 48 h, and Ts-Cc 72 h, where *T. septentrionalis* was released 24 h, 48 h, and 72 h before *C. cunea*; Cc-Ts 24 h, Cc-Ts 48 h, and Cc-Ts 72 h, where *C. cunea* was released 24 h, 48 h, and 72 h before *T. septentrionalis*. The values displayed at the base of each column indicate the number of replicates. * indicates a difference and ** indicates a strong difference (*p* < 0.05; *p* < 0.01).

The differences in the number of survival days of offspring were significant among different species of parasitoids using the same release modes, particularly in Cc-Ts 72 h. There was also highly significant variation observed in control, as well as at 24 h and 48 h time intervals, at both the order of release (*C. cunea* first or *T. septentrionalis* first) (Figure 7). When considering different release modes of parasitoids within the same species, there were highly significant differences observed between various time intervals, such as 0 h and Cc-Ts 48 h, 0 h and Cc-Ts 72 h, Ts-Cc 24 h and Cc-Ts 48 h, Ts-Cc 24 h and Cc-Ts 72 h, Ts-Cc 48 h and Cc-Ts 48 h, and Ts-Cc 48 h and Cc-Ts 72 h for *T. septentrionalis*. Additionally, significant differences were found between Ts-Cc 24 h and Ts-Cc 72 h, Ts-Cc 48 h, and Ts-Cc 72 h, as well as highly significant differences between Ts alone and Ts-Cc 24 h, Ts alone and Ts-Cc 48 h for *C. cunea* (Figure 6).

**Figure 8:**
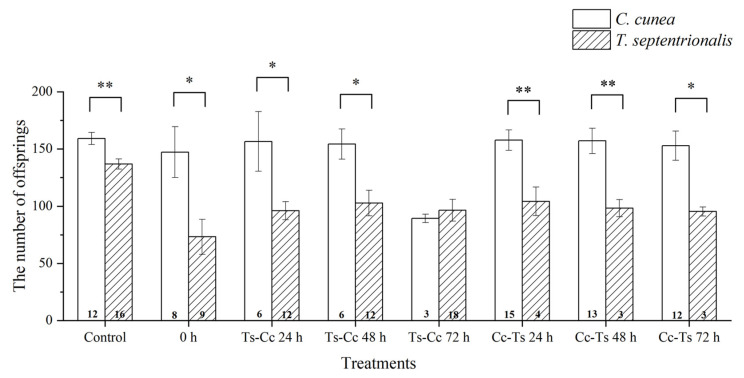
The differences in number of offspring between *Chouioa cunea* and *Tetrastichus septentrionalis* were observed in *Hyphantria cunea*. Annotation: Data are presented as mean ± SEM. Control, where only *C. cunea* or *T. septentrionalis* was released; 0 h, where *C. cunea* and *T. septentrionalis* were introduced simultaneously; Ts-Cc 24 h, Ts-Cc 48 h, and Ts-Cc 72 h, where *T. septentrionalis* was released 24 h, 48 h, and 72 h before *C. cunea*; Cc-Ts 24 h, Cc-Ts 48 h, and Cc-Ts 72 h, where *C. cunea* was released 24 h, 48 h, and 72 h before *T. septentrionalis*. The values displayed at the base of each column indicate the number of replicates. * indicates a difference and ** indicates a strong difference (*p* < 0.05; *p* < 0.01).

The differences in the number of offspring between different species of parasitoids using the same release modes were significant at 0 h, Ts-Cc 24 h, Ts-Cc 48 h, and Cc-Ts 72 h, with highly significant variation in control groups, Cc-Ts 24 h, and Cc-Ts 48 h (Figure 8). Similarly, when examining different release modes of parasitoids within the same species, significant differences were noted between Ts alone and Ts-Cc 24 h, and Ts alone and Ts-Cc 72 h, as well as highly significant differences between Ts alone and 0 h for *T. septentrionalis* (Figure 6).

## 4. Discussion

Many experiments have argued and proved that there are three reasons for competitive results: time intervals, attacks, and physiological inhibition (regulation of hemolymph or use of toxic substances) [25]. In this experiment, we mainly studied extrinsic competition and extrinsic intrinsic competition between *C. cunea* and *T. septentrionalis*. In extrinsic competition, we found that two parasitoid species did not mutually attack each other and focused on comparing their behaviors. In intrinsic competition, the species of parasitoid released first can occupy an advantage in internal competition. Regardless of the release order and oviposition interval, only one parasitoid species successfully completed its development, this result is similar to most studies [26,27]. In addition, the success rate of parasitism by *C. cunea* and *T. septentrionalis* parasitized was higher than parasitized by one species parasitoid.

The results of the competition can be attributed to various factors, including the ability to distinguish between parasitized and unparasitized hosts [28,29]. In the extrinsic competition, we focus on the search time and oviposition strategies of parasitoids. We found that both parasitoid species can parasitize a host which previously parasitized by a competitor. Since the results indicate that they cannot defeat their competitors, it can be inferred that they lack the ability to recognize parasitism. The oviposition strategies and time allocation are also factors affecting the results of the competition [30]. When there are time intervals, the oviposition frequency of *T. septentrionalis* is significantly higher than that of *C. cunea*. The oviposition frequency of parasitoid on the same host can have a significant impact on the growth, development, survival, emergence, individual size, sex ratio, and other aspects of the offspring [31]. Some studies suggest that multiple oviposition frequency and oviposition duration are forms of hyperparasitism, which helps parasitoids overcome host defenses and reduce the encapsulation effect of the host [32]. There are significant differences in host search time between two parasitoid species under single parasitism and 48 h intervals between oviposition. The primary factors influencing the search time it takes for parasitoids to oviposition encompass environmental conditions, intrinsic characteristics of parasitoids, and chemical compounds [33,34]. However, the underlying causes for this finding require more in-depth investigation. When there is an interval of oviposition between the first and second laying of eggs, parasitoids that lay their first eggs are usually more competitive than those that lay their eggs after [18].

The results of this article, we found show that the parasitoid release orders and time intervals between oviposition determine the outcome of the competition. Many studies on the biological characteristics of parasitoids’ offspring serve as a basis for evaluating parasitic effects [35,36], such as the number, female ratio, and survival days of offspring. In the experiments, the parasitoid species released 48 and 72 h in advance would have a dominant advantage in parasitic competition. This could indirectly suggest that it was the species released first that dominated, due to the time intervals. The results were similar to *Microplitis croceipes* (Cresson) and *Cardiochiles nigriceps* Viereck (both Hymenoptera: Braconidae) [37,38], *Trissolcus japonicus* (Ashmead), and *Trissolcus mitsukurii* (Ashmead) (both Hymenoptera: Scelionidae) [39], which proved that the offspring always tend to favor the first species in terms of parasitization. According to the related research on the developmental duration of *C. cunea* and *T. septentrionalis* [23,24], the reason for the species released first dominating the competition may be the duration of embryological development.

The female ratio of parasitoid offspring under different treatments did not change significantly, which may be related to the stability of their sex determination mechanism, resource utilization, and oviposition strategies in response to harsh environments. Females of parasitoids compete for hosts, and this competitive interaction can affect the viability and sex ratio of offspring and favor the occurrence of multiparasitism [40]. Stable sex ratio inheritance may be the result of the evolution of two parasitic wasps suitable for population reproduction in a long-term competitive environment, and the specific reasons remain to be studied. Endoparasitoids usually optimize inclusive fitness due to these reasons, and the survival time is often severely limited. Thus, the survival days of the offspring of parasitoids are an important indicator to determine the nature of the competition [41].

Survival days were also important for describing differences in the state of the host and the effects of the parasitism [42]. We found variation in the results based on different conditions, and the survival days also showed significant differences and were used as an index for identifying parasitic effects. The survival time of offspring of *C. cunea* and *T. septentrionalis* changed under different conditions, possibly due to the intense competition between *C. cunea* and *T. septentrionalis*. The number of survival days of offspring in *Campoletis sonorensis* (Cameron) (Hymenoptera, Ichneumonidae), *Microplitis demolitor* Wilkinson (Hymenoptera: Braconidae), and *Microplitis croceipes* (Cresson) (Hymenoptera: Braconidae) under different conditions also changed [43].

The number of offspring was influenced by the demand for parasitoids for their host [44]. Due to the limited host resources, the competition may rise through the developmental needs of parasitoids after laying eggs inside the host. The number of offspring of *T. septentrionalis* is significantly lower than that when only releasing *T. septentrionalis*, the lowest case occurs in the 0 h time interval. In previous experiments with parasitoids, a certain species consistently dominated regardless of the time intervals and sequences tested. For instance, *Ooencyrtus telenomicida* (Vassiliev) (Hymenoptera: Encyrtidae) tended to dominate *Trissolcus basalis* (Wollaston) (Hymenoptera: Scelionidae), with *O. telenomicida* ovipositing after *T. basalis* and eventually taking a dominant position [19]. A similar trend was observed between *Tamarixia lyciumi* Yang (Hymenoptera: Eulophidae) and *Psyllaephagus arenarius* Trjapitzin (Hymenoptera: Encyrtidae) [45]. However, our experimental result differed different from these findings, it may be due to the comparable parasitic capabilities of the two parasitic wasps. The results of *C. cunea* may not experience a significant change in quantity due to its physiological and other advantages, but after releasing *T. septentrionalis* for 72 h, the development of offspring of *T. septentrionalis* approaches maturity, leading to more intense competition and the death of a large number of offspring of *C. cunea*. However, this hypothesis remains to be confirmed in further study.

## 5. Conclusions

In experiments, studies that focus on extrinsic (search time, oviposition frequency, and oviposition duration) and intrinsic (number, female ratio, and survival days of offspring) factors can assist in the evaluation and planning of the use of biological agents. Understanding the emergence of offspring from biological agents can help reduce the frequency of parasitoids released in the field. By examining the interactions between parasitoids, this research provides a foundation for studying the interaction mechanism of natural enemies of *H. cunea* in the field. Using two parasitoid species results in a higher parasitism rate on the target host than using one parasitoid species. This study on competition between the two dominant parasitic natural enemies of *H. cunea* in the Dandong area, Liaoning Province, provides a theoretical basis for the future application of multiple natural enemies for the biological control of *H. cunea*.

## Figures and Tables

**Table 1:** Mortality, parasitism, and host emergence ratio of *Chouioa cunea* and *Tetrastichus septentrionalis* were observed in *Hyphantria cunea* in intrinsic competition at different time intervals between oviposition and release sequences.

Treatment	*n*	Mortality (%)	Parasitism (%)	Host Emergence (%)	Percentage of Adult Emergence (%)	
					*C. cunea*	*T. septentrionalis*
Only Cc	25	20	48	32	100	-
Only Ts	25	20	64	16	-	100
Ts-Cc 0 h	25	12	68	20	47.06	52.94
Ts-Cc 24 h	25	12	72	16	33.33	66.67
Ts-Cc 48 h	25	12	72	16	33.33	66.67
Ts-Cc 72 h	25	16	84	0	14.3	85.7
Cc-Ts 24 h	25	8	76	16	78.95	21.05
Cc-Ts 48 h	25	24	64	12	81.25	18.75
Cc-Ts 72 h	25	28	60	12	80	20
Ts and Cc	175	16	70.96	13.14	50.81	49.19

Annotation: Only Cc and Only Ts, where only *C. cunea* or *T. septentrionalis* was released; Ts-Cc 0 h, where *C. cunea* and *T. septentrionalis* were introduced simultaneously; Cc-Ts 24 h, 48 h, and 72 h, where *C. cunea* was released 24 h, 48 h, and 72 h first. Ts-Cc 24 h, 48 h, and 72 h, where *T. septentrionalis* was released 24 h, 48 h, and 72 h first. Ts-Cc was all treatments that release two species of parasitoids (Ts-Cc 0, 24, 48, 72 h, and Cc-Ts 24, 48, 72 h). n represents the number of repetitions.

## Data Availability

All relevant data are included in the article.
